# Medicare Opt-Out Trends Among Dermatologists May Reflect Systemic Health Policy: Cross-sectional Analysis

**DOI:** 10.2196/42345

**Published:** 2022-11-08

**Authors:** Aneesh Agarwal, Joseph Han, Yen Luu, Nicholas Gulati

**Affiliations:** 1 Department of Dermatology Icahn School of Medicine at Mount Sinai New York, NY United States; 2 School of Medicine University of Missouri-Kansas City Kansas City, MO United States

**Keywords:** Medicare, dermatology, opt out, private contracting, CMS, health policy, insurance, health coverage, Medicaid

## Abstract

**Background:**

Provider opt-out of accepting Medicare insurance is a nationally tracked metric by the Centers for Medicare & Medicaid Services (CMS) for all physicians, including dermatologists. Although this usually only consists of a small number of providers, the magnitude of opting out has varied historically, often tracing changes in systemic health care policy.

**Objective:**

In this paper, we explored dermatologist opt-out data since 2001, as reported by the CMS, to characterize trends and provide evidence that shifts in provider opt-out may represent a potential indicator of the state of health policy and possible needs for reform as it pertains to Medicare.

**Methods:**

The publicly available Opt Out Affidavits data set, available from the CMS, was evaluated for providers in all dermatologic specialties from January 1, 2001, to May 27, 2022.

**Results:**

There were a total of 196 dermatology opt-outs in the overall period, with the largest spike being 33 providers in 2016, followed by generally consistent decreases through 2021. In the most recent 12 months of data, the number of new monthly opt-outs from January 2022 to May 2022 was significantly higher than that of the trailing 7 months of 2021 (*P*=.03).

**Conclusions:**

Despite decreasing numbers of dermatologist opt-outs in the late-2010s, 2022 was marked by a significant increase in opt-outs. The reduced acceptance of Medicare by dermatologists may present risks to care access, so it is important to frequently assess physician opt-out data and changes over time.

## Introduction

Private contracting with Medicare patients is a practice associated with provider “opt-out” from the federal program, where billing and collecting from Medicare is precluded; although the impact of dermatologist opt-out likely varies based on factors such as practice type, provider density, and population composition, fewer physicians accepting Medicare inherently presents greater risks for care access, especially in remote, low-income, or population-sparse areas [1].

Due to the Medicare program’s role in providing broad access to care, it is important to explore characteristics associated with provider Medicare opt-out and trends over time to assess potential impacts on aspects of care delivery. Although literature on opting out is limited and the practice is infrequent [2,3], trends among provider opt-out may be revelatory of systemic issues such as complex Medicare reimbursement [1], bureaucratic intricacies, and prolonged accounts receivable periods, which can strain practitioners [4]. Therefore, assessing national metrics such as Medicare opt-out may also provide insights into health policy and systemic changes that shape Medicare provider participation.

## Methods

This cross-sectional analysis evaluates publicly available data from the Opt Out Affidavits data set available from the Centers for Medicare & Medicaid Services, comprehensive of all 50 states and the District of Columbia. We included all entries for physicians indicating dermatologic specialties over the total available period (from January 1, 2001, to May 27, 2022).

## Results

**Figure 1:**
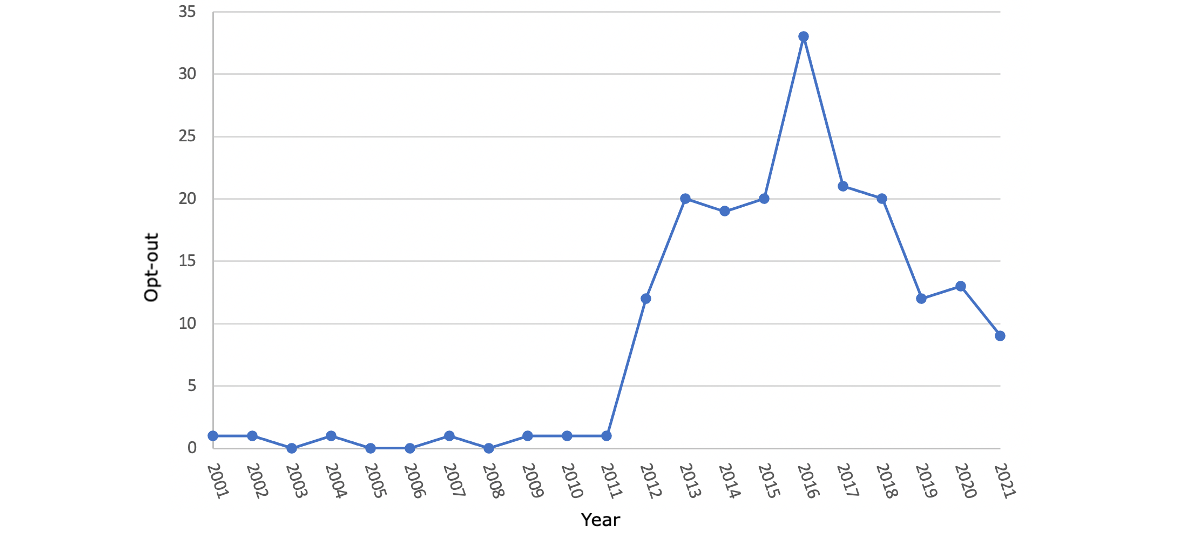
Annual number of new dermatologist opt-outs as reported by the Centers for Medicare & Medicaid Services.

There were 196 providers in the overall period who opted out of Medicare. From 2001 to 2011, annual opt-outs were ≤1. In 2012, twelve new providers opted out, followed by annual increases and a peak of 33 in 2016. After 2016, new opt-outs generally decrease by up to 12 providers annually, with a maximum decrease of 40% (8/20) from 2018-2019 (Figure 1). In 2021, there were 9 new opt-outs, and there were 10 in the first 5 months of 2022. Considering the most recent 12 months, the number of new monthly opt-outs for the first 5 months of 2022 (mean 2.0) was significantly higher than that of the trailing 7 months of 2021 (mean 0.57; *P*=.03). In the entire period, 112 (N=196, 57.1%) providers were located in New York, Texas, or California.

## Discussion

Overall, 196 (1.8%) dermatologists out of 11,003 total practicing dermatologists in the United States [3] opted out of Medicare. The majority of opt-outs were seen in New York, Texas, and California; although some of these opt-out providers are located in cities with populations lower than 10,000, all are in localities comprising statistical metropolitan areas, suggesting that there is likely still reasonable access to alternate avenues of care for Medicare beneficiaries in these areas. Opt-outs were uncommon until 2012, but the period from 2012 to 2016 represented the largest recorded spike.

Given that provider enrollment for participation in the Medicare program, or “opting in,” is a relatively uncomplicated process consisting of a 1-time application, other persistent systemic issues may have relevance to the mid-2010s shift. Rising practice operational expenses [1], complex compliance or regulatory requirements, and uncertainties from delayed payments [3,4], along with resource-constraining policies such as prior authorizations, can make it challenging for providers to effectively deliver patient-centric care [5]. The mid-2010s surge may be explained by heightened consolidation, as 15% of clinic acquisitions among private equity groups from 2014 to 2016 were dermatology clinics [3]. Greater prevalence of large group practices can present difficulty for independent practitioners to negotiate with insurers [3] and remain economically viable if Medicare comprises a large portion of their payer mix given the associated administrative challenges [5]. Another possible contributor to the 2016 spike may be the Medicare Access and CHIP (Children’s Health Insurance Program) Reauthorization Act of 2015; although beneficial in promoting patient-centric care, it may be accompanied by a higher risk exposure for providers and additional administrative strain [6]. Further investigation and provider surveying are needed to determine which specific issues are driving the described patterns in provider opt-out, since it is unclear whether the primary catalyst for provider opt-out is economic, logistic, or administrative factors. Although the reduction in dermatology opt-outs during the late-2010s likely represents a positive shift for patients and providers, the latest data show a significant monthly increase in opt-out providers, which should be monitored to ensure optimal care access for communities. Limitations of this analysis include the lack of commercial insurance opt-out data, absent information on nonphysician provider statuses, and unavailable information around reopting into Medicare or those who retired with opt-out status.

In an indirect manner, Medicare opt-out has been previously proposed as a figurative voice for providers to express sentiments about reimbursement policy [1] and may implicitly represent the impacts of other policy challenges on the state of practice. Additionally, the implications of physician opt-out can be broad, where individuals served by Medicare in certain localities may experience inadequate access to care and poorer health outcomes with increasing provider opt-out. As a result, trends in Medicare opt-out should be followed closely to evaluate possible needs to review or refine systemic dermatologic health policy in favor of both patients and providers.

## Abbreviations

CHIP: Children’s Health Insurance Program
CMS: Centers for Medicare & Medicaid Services
